# Supplementary material for: Mutations in PCYT2 disrupt etherlipid biosynthesis and cause a complex hereditary spastic paraplegia
Source: Brain. 2019 Oct 22;142(11):3382–97. doi: 10.1093/brain/awz291 (PMC6821184; doi:10.1093/brain/awz291)
Supplement: awz291_Supplementary_Data [file awz291_supplementary_data.zip › awz291-Suppl_data/Supplementary_Data4.zip!/Supplementary information 4 - plasma lipidomics.zip!/Plasma lipidomics/Control vs Patient1/Statistical plots and data/PA species/Heatmap.pdf]

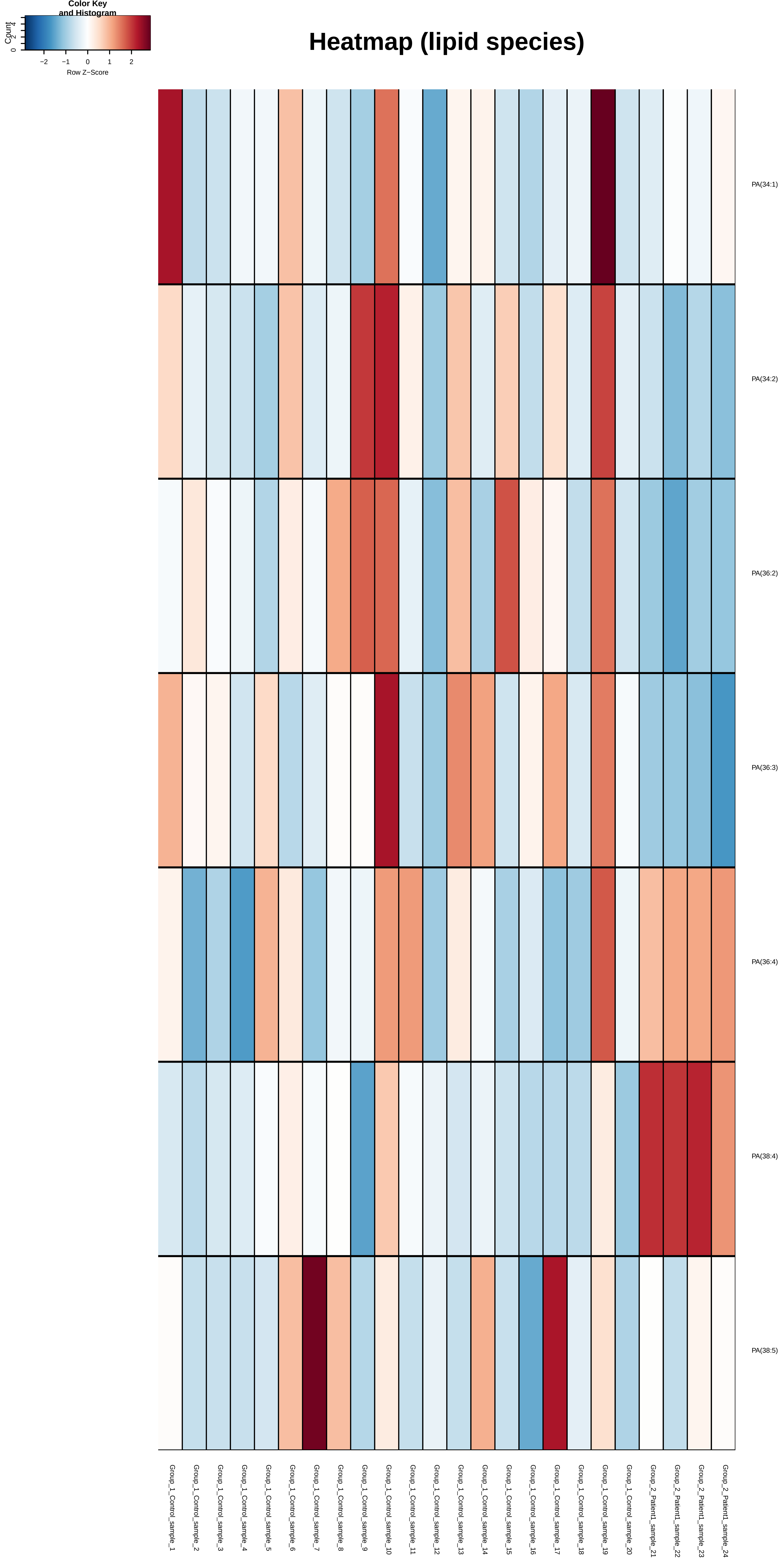
Color Key
and Histogram
Count
0
2
4
−2
−1
0
1
2
Row Z−Score
Heatmap (lipid species)
PA(34:1)
PA(34:2)
PA(36:2)
PA(36:3)
PA(36:4)
PA(38:4)
PA(38:5)
Group_1_Control_sample_1
Group_1_Control_sample_2
Group_1_Control_sample_3
Group_1_Control_sample_4
Group_1_Control_sample_5
Group_1_Control_sample_6
Group_1_Control_sample_7
Group_1_Control_sample_8
Group_1_Control_sample_9
Group_1_Control_sample_10
Group_1_Control_sample_11
Group_1_Control_sample_12
Group_1_Control_sample_13
Group_1_Control_sample_14
Group_1_Control_sample_15
Group_1_Control_sample_16
Group_1_Control_sample_17
Group_1_Control_sample_18
Group_1_Control_sample_19
Group_1_Control_sample_20
Group_2_Patient1_sample_21
Group_2_Patient1_sample_22
Group_2_Patient1_sample_23
Group_2_Patient1_sample_24
